# Epidemiologic trends and survival of early-onset gastroenteropancreatic neuroendocrine neoplasms

**DOI:** 10.3389/fendo.2023.1241724

**Published:** 2023-08-28

**Authors:** Hailing Yao, Gengcheng Hu, Chen Jiang, Mengke Fan, Lanlai Yuan, Huiying Shi, Rong Lin

**Affiliations:** ^1^ Department of Gastroenterology, Union Hospital, Tongji Medical College, Huazhong University of Science and Technology, Wuhan, China; ^2^ Department of Otorhinolaryngology, Union Hospital, Tongji Medical College, Huazhong University of Science and Technology, Wuhan, China

**Keywords:** early-onset gastroenteropancreatic neuroendocrine neoplasms, epidemiology, incidence, mortality, survival

## Abstract

**Background:**

The epidemiologic trends and survival related to early-onset gastroenteropancreatic neuroendocrine neoplasms (GEP-NENs) have not been well explored.

**Methods:**

Trends in the incidence and incidence-based mortality of early-onset GEP-NENs between 1975 and 2018 were obtained from the Surveillance, Epidemiology, and End Results database, and were stratified by age, sex, race, tumor site, stage, and grade. Associated population data were used to determine overall survival (OS) and independent prognostic factors for patients with early-onset GEP-NENs.

**Results:**

A total of 17299 patients diagnosed with early-onset GEP-NENs were included in this study. Results revealed an increase in the incidence (5.95% per year, 95% confidence interval (CI), 5.75-6.14%) and incidence-based mortality (4.24% per year, 95% CI, 3.92-4.56%) for early-onset GEP-NENs from 1975 to 2018, with higher rates of increase than those of later-onset GEP-NENs (incidence: 4.45% per year, 95% CI, 4.38-4.53; incidence-based mortality: 4.13% per year, 95% CI, 3.89-4.37; respectively). Increases in incidence were observed across all age, races, tumor sites, grades, and stages, except for patients with unknown stage. Compared to those with later-onset GEP-NENs, a higher proportion of female gender (54.5% vs. 49.0%, *p <*0.001), well-differentiated tumor (31.1% vs. 28.0%, *p <*0.05), and localized disease (55.2% vs. 46.7%, *p <*0.05) were observed in the cohort of patients with early-onset GEP-NENs. Moreover, early-onset GEP-NENs exhibited a superior overall survival in comparison to later-onset GEP-NENs, irrespective of tumor site, grade, or stage (*p <*0.0001). Multivariable survival analysis identified that race, marital status, stage, grade, chemotherapy, and primary site were significantly correlated with OS in individuals with early-onset GEP-NENs.

**Conclusions:**

The incidence and incidence-based mortality rates of early-onset GEP-NENs have steadily increased over time, with higher rates of increase than those of later-onset GEP-NENs. The clinical characteristics and survival were different between early-onset and later-onset GEP-NENs groups. Race, marital status, stage, grade, chemotherapy, and primary site were independent prognostic factors for early-onset GEP-NENs. Further investigations are warranted to better understand the characteristics of this disease subgroup.

## Introduction

Recent evidence has revealed a concerning trend indicating an increase in the incidence of early-onset cancer (cancers diagnosed <50 years of age) (1). Reported people aged 25-49 years had the largest proportional rise in the incidence of all cancers in the UK, representing a 22% increase from 1993 to 2018 (2). The US National Cancer Institute has highlighted the “early-onset cancer epidemic” as a research priority in its “Provocative Questions” for 2020-2021 (3). Globally, cancer was the fourth leading cause of death among individuals aged 15-39 years in 2019 (4). In addition, studies have demonstrated that survivors of early-onset cancers are susceptible to various health problems such as second malignant neoplasms, cardiovascular diseases, gonadal dysfunction, and adverse pregnancy outcomes (5–7). Early-onset cancer has become a considerable concern among experts worldwide.

A large, population-based study reported an increase in the incidence rate of gastroenteropancreatic neuroendocrine neoplasms (GEP-NENs) among adults aged less than 50 years from 1975 to 2012 (8). The reasons for this rising phenomenon are unknown. One retrospective study indicated patients aged 60 years or younger had a 2-fold higher risk of developing NENs than those aged over 60 years (9). In a large cohort of 2.3 million Israeli adolescents, Katz et al. reported that increased height and weight were correlated with the risk of gastric NENs (10). A Swedish study reported that socioeconomic status had an impact on the development of NENs (11). Multiple studies showed a family history of cancer and diabetes may be associated with the development of GEP-NENs (12, 13). Given the rising incidence of early-onset GEP-NENs, patients with early-onset GEP-NENs represent a small but increasing and relevant population that needs greater attention and further study. However, little is known about the epidemiology and survival of patients with early-onset GEP-NENs due to the rarity and indolent clinical behaviors.

Therefore, our study aimed to investigate the incidence, mortality, survival, and prognostic factors of early-onset GEP-NENs obtained from a large population-based database in the US.

## Methods

### Data source

The Surveillance, Epidemiology, and End Results (SEER) database is a national cancer database that reports historical population-based incident malignancies from target geographic areas, covering nearly 35% of the US population. The SEER database was utilized to identify patients diagnosed with primary GEP-NENs between 1975 and 2018. Individuals with early-onset GEP-NENs were defined as those with an age at diagnosis of less than 50 years, while the remaining were defined as later-onset GEP-NENs. Cancers typically affect individuals aged 50 and older, and the historical guidelines recommended screening for gastrointestinal cancers starting at 50 years (14–16). Furthermore, the age of 50 years has been broadly accepted as the threshold for defining early-onset cancers in the medical community, facilitating consistent comparisons across various studies (17–19). Consequently, we selected 50 years as the threshold for distinguishing between early-onset and later-onset GEP-NENs.

To maximize the representativeness of our study, we used SEER 8 to calculate the 1975-1999 trends and the SEER 17 database to calculate the 2000-2018 trends (20, 21). SEER*Stat software (version 8.4.0.1) was employed to compute the incidence and mortality rates. All rates reported in the SEER database are per 100 000 population and age-adjusted to the 2000 US standard population. Incidence-based mortality rates were calculated as the number of early-onset GEP-NENs deaths among cases diagnosed in the SEER registries, and the mortality data sourced from the US Mortality Files, National Center for Health Statistics, Centers for Disease Control and Prevention.

Histologic codes from the *International Classification of Diseases (ICD) for Oncology, Third Edition* (8013, 8150-8156, 8240-8246, 8249, 8574, and 9091; see **Supplemental Table 1**) and site codes (stomach, 160-169; small intestine, 170 to 179; pancreas, 250-259; appendix, 181; colon, 180 and 182 to 189; rectum, 199 and 209) were utilized to identify all GEP-NENs patients from 1975 to 2018. Patients with other secondary cancers were excluded. The data are publicly available and do not require ethical approval for analysis. The authors obtained limited-use data approval from SEER.

Cause of death was categorized by the *ICD* 10^th^ code. We grouped patients into three cause of death categories: death from early-onset GEP-NENs, death from other cancers, and death from noncancer causes.

### Demographic characteristics

Patient demographic and clinical characteristics, including age at diagnosis, race, sex, marital status, primary tumor site, grade, SEER stage, treatment (surgery, chemotherapy, and radiotherapy), cause of death, survival time, and outcome were extracted. In terms of tumor grade, well-differentiated, moderately differentiated, poorly differentiated, and undifferentiated were defined as grade I, II, III, and IV, respectively. Grade III and grade IV are combined into one category for all analyses. The survival time was calculated as the time from the diagnosis of cancer to the death of the patients due to any cause or the end of follow-up.

### Statistical analysis

The Joinpoint Regression Program (version 4.4.0) was used to calculate the long-term trends in incidence and mortality, with trends expressed as annual percent changes (APCs) and 95% confidence interval (CI). T-test was used to determine whether APCs were significantly different from zero. Kaplan–Meier (K-M) analysis, and Cox proportional hazards regression models were calculated with R studio (version 4.1.0), and the two-tailed log-rank test was used to assess the difference in survival. Statistical significance was assessed at *p <*0.05, and all hypotheses were two-sided.

## Results

### Baseline characteristics

A total of 80581 patients diagnosed with GEP-NENs were identified from the SEER database from 1975 to 2018, of whom 17258 patients were early-onset GEP-NENs, and the remaining 63323 patients were later-onset GEP-NENs. The demographic and clinical characteristics of patients with GEP-NENs are shown in **Table 1**. In the whole study population, the majority of patients were White, married, had localized disease, and received surgical treatment. There were significant differences in patient characteristics between the two groups. Compared to later-onset GEP-NENs, early-onset GEP-NENs exhibited a higher proportion of female gender (54.5% vs. 49.0%, *p <*0.001), well-differentiated tumor (31.1% vs. 28.0%, *p <*0.05), and localized disease (55.2% vs. 46.7%, *p <*0.05). In addition, early-onset GEP-NENs were more frequently located in the rectum (27.1% vs. 25.4%, *p <*0.05) and appendix (25.3% vs. 6.4%, *p <*0.05) in comparison to later-onset GEP-NENs.

**Table 1:** Baseline clinicopathological Characteristics of gastroenteropancreatic neuroendocrine tumors.

Characteristics	Incidence			Incidence-based mortality		
	Early-onset	Later-onset	*p* value	Early-onset	Later-onset	*p* value
**Overall**	17258 (100)	63323 (100)		3110 (100)	26102 (100)	
**Age at diagnosis**			–			–
0-14	327 (1.9)	–		11 (0.4)	–	
15-24	1414 (8.2)	–		80 (2.6)	–	
25-34	2963 (17.2)	–		362 (11.6)	–	
35-44	6617 (38.3)	–		1267 (40.7)	–	
45-49	5937 (34.4)	–		1390 (44.7)	–	
**Sex**			<0.001			0.296
Male	7845 (45.5)	32280 (51.0)		1638 (52.7)	14006 (53.7)	
Female	9413 (54.5)	31043 (49.0)		1472 (47.3)	12096 (46.3)	
**Race**			<0.001			<0.001
White	12617 (73.1)	47427 (74.9)		2337 (75.1)	20948 (80.3)	
Black	2586 (15.0)	9497 (15.0)		523 (16.8)	3538 (13.6)	
AI/AN	161 (0.9)	393 (0.6)		30 (1.0)	144 (0.6)	
AP	1347 (7.8)	4663 (7.4)		213 (6.8)	1434 (5.5)	
Unknown	547 (3.2)	1343 (2.1)		7 (0.2)	38 (0.1)	
**Marital status**			<0.001			<0.001
Single	5411 (31.4)	7774 (12.3)		883 (28.4)	2984 (11.4)	
Married	8732 (50.6)	35971 (56.8)		1683 (54.1)	14411 (55.2)	
Sep/Div/Wid	1156 (6.7)	13041 (20.6)		273 (8.8)	7113 (27.3)	
Unknown	1959 (11.4)	6537 (10.3)		271 (8.7)	1594 (6.1)	
**Grade**			<0.001			<0.001
I	5367 (31.1)	17722 (28.0)		445 (14.3)	4373 (16.8)	
II	1229 (7.1)	4478 (7.1)		237 (7.6)	1707 (6.5)	
III+IV	675 (3.9)	4421 (7.0)		502 (16.1)	3729 (14.3)	
Unknown	9987 (57.9)	36702 (58.0)		1926 (61.9)	16293 (62.4)	
**Stage**			<0.001			<0.001
Localized	9527 (55.2)	29563 (46.7)		555 (17.8)	6988 (26.8)	
Regional	2785 (16.1)	11885 (18.8)		426 (13.7)	4688 (18.0)	
Distant	2228 (12.9)	11744 (18.5)		1282 (41.2)	8241 (31.6)	
Unknown	2718 (15.7)	10131 (16.0)		847 (27.2)	6185 (23.7)	
**Surgery**			<0.001			<0.001
Yes	14071 (81.5)	46420 (73.3)		1891 (60.8)	16622 (63.7)	
No	2978 (17.3)	16134 (25.5)		1168 (37.6)	9186 (35.2)	
Unknown	209 (1.2)	769 (1.2)		51 (1.6)	294 (1.1)	
**Chemotherapy**			0.005			<0.001
Yes	1649 (9.6)	6516 (10.3)		1097 (35.2)	4808 (18.4)	
No/Unknown	15609 (90.4)	56807 (89.7)		2013 (64.7)	21294 (81.6)	
**Radiation**			0.043			<0.001
Yes	342 (2.0)	1416 (2.2)		225 (7.2)	1071 (4.1)	
No/Unknown	16916 (98.0)	61907 (97.8)		2885 (92.8)	25031 (95.9)	
**Primary sites**			<0.001			<0.001
Appendix	4358 (25.3)	4053 (6.4)		329 (10.6)	1180 (4.5)	
Colon	1018 (5.9)	6436 (10.2)		366 (11.8)	3872 (14.8)	
Rectum	4683 (27.1)	16078 (25.4)		471 (15.1)	3762 (14.4)	
Small intestine	2989 (17.3)	19376 (30.6)		615 (19.8)	8968 (34.3)	
Stomach	1425 (8.3)	6568 (10.4)		270 (8.7)	2912 (11.2)	
Pancreas	2785 (16.1)	10812 (17.1)		1059 (34.1)	5408 (20.7)	

AI/AN, American Indian/Alaska Native; AP, Asian or Pacific Islander; Sep/Div/Wid, Separated/Divorced/Widowed.

Among the participants included in our study, a total of 29212 deaths occurred, including 3110 patients with early-onset GEP-NENs and the remaining 26102 patients with later-onset GEP-NENs. The majority of patients who died were male, White, married, and had distant metastasis. In terms of tumor location, patients with early-onset GEP-NENs located in the pancreas (34.1%) were more likely to die, whereas patients with later-onset GEP-NENs located in the small intestine (34.3%) were more likely to die.

### Disparities in the incidence, mortality, and survival between early-onset and later-onset GEP-NENs

**Figure 1:**
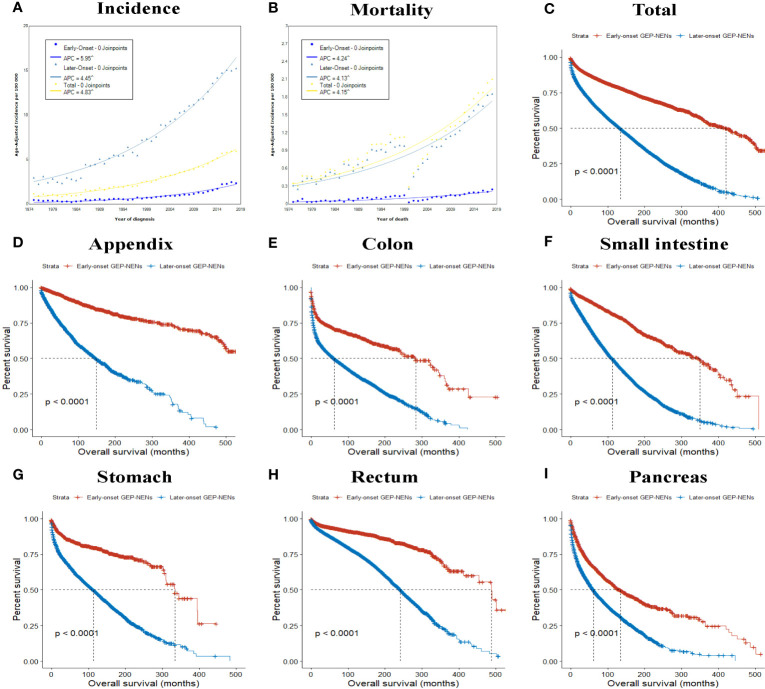
Incidence **(A)** and incidence-based mortality **(B)** trends of GEP-NENs over time (1975–2018) in the United States according to age. **(C–I)** The overall survival between early-onset GEP-NENs and later-onset GEP-NENs by primary site, **(C)** total, **(D)** appendix, **(E)** colon, **(F)** small intestine, **(G)** stomach, **(H)** rectum, **(I)** pancreas.

As shown in **Figure 1A**, the incidence of all GEP-NEN cases increased throughout the study period (APC, 4.83; 95% CI, 4.76-4.89). The incidence of early-onset GEP-NENs ranged from 0.46 to 2.34 per 100 000 persons from 1975 to 2018, while the incidence of later-onset GEP-NENs ranged from 2.97 to 15.24 per 100 000 persons. The incidence of early-onset GEP-NENs (APC, 5.95; 95% CI, 5.75-6.14) exhibited a greater increase than that of later-onset GEP-NENs (APC, 4.45; 95% CI, 4.38-4.53).

Regarding incidence-based mortality, there was a steady increase in overall GEP-NENs (from 0.35 per 100 000 in 1975 to 2.10 per 100 000 in 2018), increasing by 4.15% (95% CI, 3.90-4.40) per year (**Figure 1B**). Similar to the trend observed in the incidence, the increase in incidence-based mortality was higher for early-onset GEP-NENs (APC, 4.24; 95% CI, 3.92-4.56) than for later-onset GEP-NENs (APC, 4.13; 95% CI, 3.89-4.37).

The results of K-M survival analysis indicated that the prognosis of early-onset GEP-NENs was significantly superior to that of later-onset GEP-NENs (**Figure 1C**), regardless of the tumor site (**Figures 1D–I**), stage (**Supplementary Figures 1A–D**), or grade (**Supplementary Figures 1E–H**) (all p <0.0001).

### Incidence of early-onset GEP-NENs by age, sex, race, tumor site, stage, and grade

**Figure 2:**
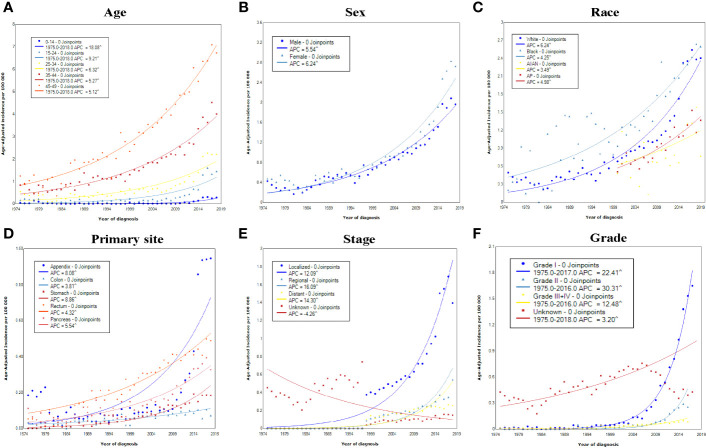
Incidence rates of early-onset GEP-NENs over time (1975–2018) in the United States by **(A)** age, **(B)** sex, **(C)** race, **(D)** primary site, **(E)** SEER stage, and **(F)** grade.

Age-specific incidence rates for early-onset GEP-NENs were calculated, and the results showed that the incidence among patients aged 0-14 years increased most significantly, from 0.04 in 1975 to 0.28 per 100 000 persons in 2018 (APC, 18.08; 95% CI, 14.91-21.34) (**Figure 2A**). Regarding sex, female patients exhibited a higher absolute incidence (2.73 per 100,000 persons) and rate of increase (APC, 6.24; 95% CI, 5.99-6.48) than male patients (1.97 per 100 000 persons; APC, 5.54; 95% CI, 5.38-5.70) (**Figure 2B**). Among race groups, the long-term trends suggested that the incidence increased the most among White patients (APC, 6.24; 95% CI, 6.01-6.46) (**Figure 2C**).

The trends in the incidence of early-onset GEP-NENs were further explored for different primary sites, stages, and grades. The increase in the incidence occurred across all tumor sites, with APCs for various sites ranging from 3.81 (95% CI, 2.98-4.65) for the colon to 8.86 (95% CI, 7.83-9.91) for the stomach (**Figure 2D**). Significant increases in the incidence over time were observed in localized, regional, and distant disease (**Figure 2E**), with the incidence of regional early-onset GEP-NENs showing the greatest increase (APC, 16.09; 95% CI, 13.82-18.40), while the incidence in the unknown stage group decreased by 4.26 (95% CI, 3.79–6.07). As for different grades, the incidence increased the most for grade II early-onset GEP-NENs, from 0.01 per 100 000 persons to 0.26 per 100 000 persons (APC, 30.31; 95% CI, 27.13-33.56) (**Figure 2F**).

### Incidence-based mortality rates and causes of death of early-onset GEP-NENs

**Figure 3:**
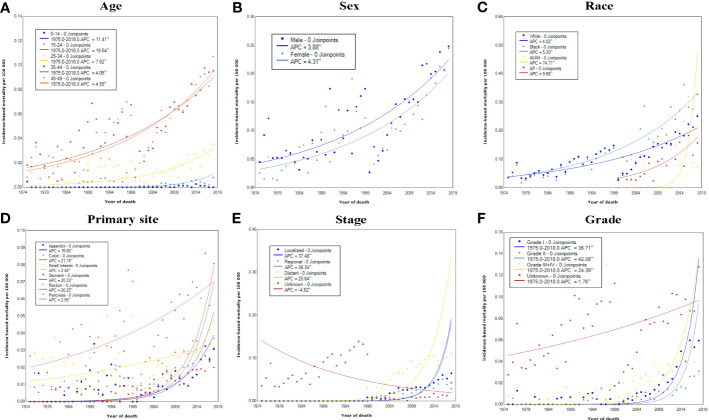
Incidence-based mortality rates of early-onset GEP-NENs over time (1975–2018) in the United States by **(A)** age, **(B)** sex, **(C)** race, **(D)** primary site, **(E)** SEER stage, and **(F)** grade.

The incidence-based mortality of early-onset GEP-NENs increased, rising from 0.04 per 100 000 in 1975 to 0.25 per 100 000 in 2018 (**Figure 1B**). Positive APCs in the incidence-based mortality rates were observed across all subgroups categorized by sex, race, tumor site, stage, and grade (**Figures 3A–F**). The only exception was among patients with unknown stage, which showed a significant decrease in mortality rates by 4.52% (95% CI, 3.74-5.29).

**Figure 4:**
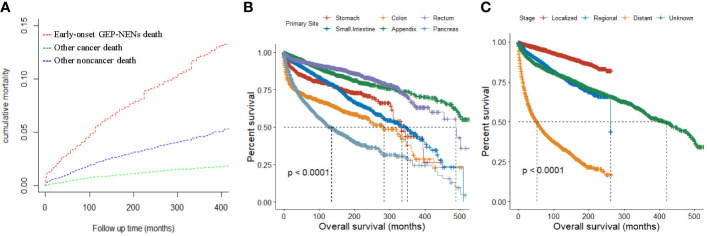
**(A)** Cumulative mortality for all causes of death in early-onset GEP-NENs patients. **(B, C)** Survival analysis of early-onset GEP-NENs by primary site **(B)** and SEER stage **(C)**.

**Figure 4A** displayed the cumulative mortality attributed to diverse causes of death among patients with early-onset GEP-NENs. At 400 months of follow-up, the cumulative mortality for early-onset GEP-NENs, other cancers, and other noncancer deaths were 13.14%, 1.80%, and 5.09%, respectively.

### Survival

Overall, the median overall survival (OS) for all patients was 420 months (range, 383-454 months). Patients with early-onset GEP-NENs in the appendix (median OS, not reached [NR]) or rectum (median OS, 488 months) had the best median OS among the tumor site groups, while patients with early-onset GEP-NENs in the pancreas (134 months) had the worst median OS (*p <*0.0001) (**Figure 4B**). Patients with localized early-onset GEP-NENs had the best median OS (NR) compared to those with regional (261 months) and distant (53 months) early-onset GEP-NENs (*p <*0.0001) (**Figure 4C**).

Survival patterns were further assessed based on primary sites and stages, as presented in **Supplemental Table 2**. Among patients with localized early-onset GEP-NENs, the median OS was not reached for all sites. Regional early-onset GEP-NENs in the rectum exhibited the poorest median OS (147 months), followed by the pancreas (261 months), while the median OS for other sites was not reached. For patients with distant early-onset GEP-NENs, the small intestine demonstrated the most favorable median OS (174 months), whereas the colon (13 months), stomach (14 months), and rectum (15 months) had the worst median OS (*p <*0.0001).

**Figure 5:**
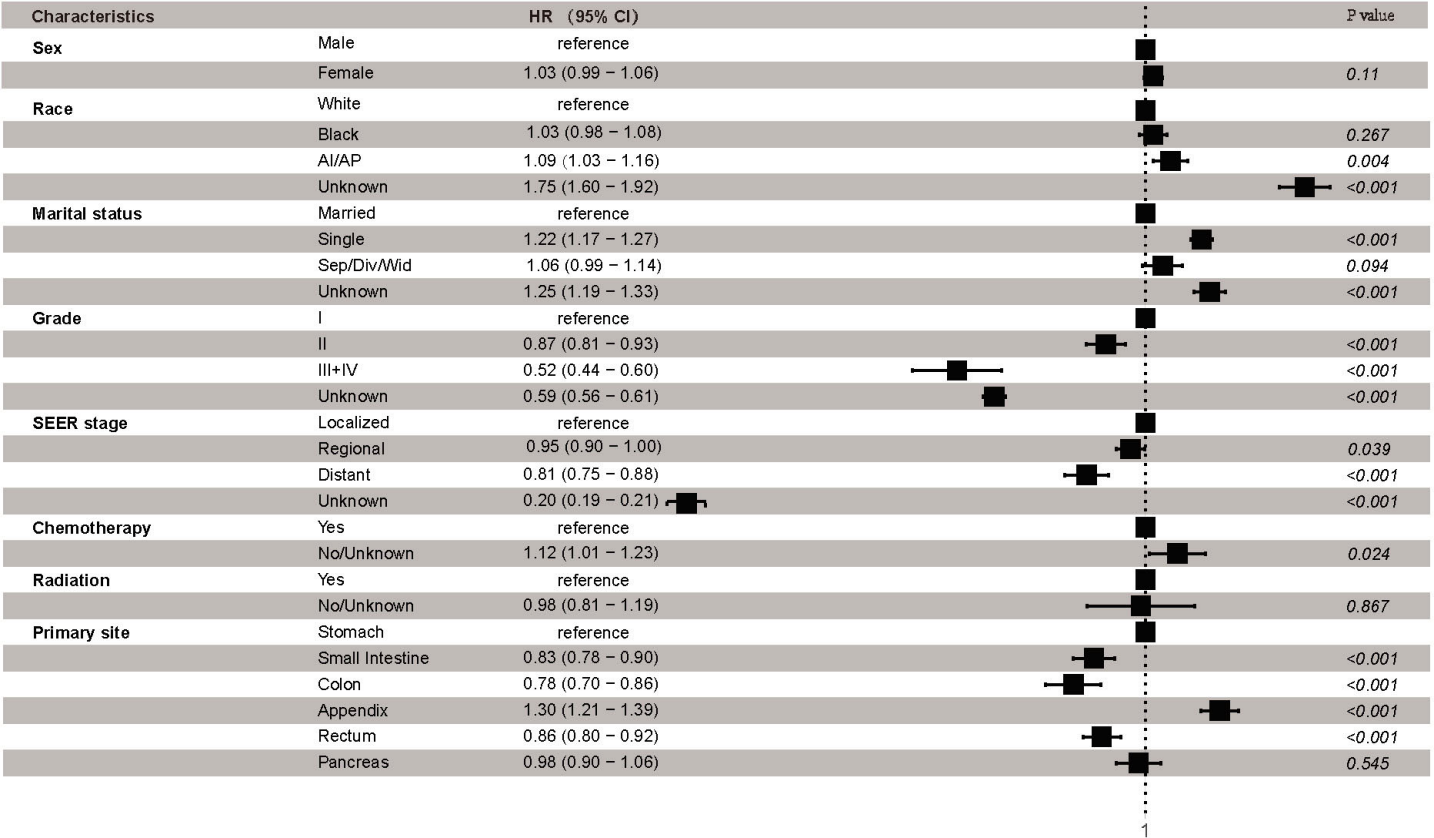
Multivariable regression analysis for early-onset GEP-NENs.

Finally, Cox proportional hazards regression was conducted to explore the independent prognostic risk factors for patients with early-onset GEP-NENs. The results of the univariate analysis revealed that sex, race, marital status, grade, SEER stage, radiation, chemotherapy, and primary site were significantly associated with OS (**Supplemental Table 3**). Multivariable survival analysis identified that race, marital status, grade, SEER stage, chemotherapy, and primary site were independent prognostic factors of early-onset GEP-NENs (**Figure 5**). Compared to early-onset GEP-NENs located in the stomach, those located in the small intestine (HR, 0.83; 95% CI, 0.78-0.90; *p <*0.001), colon (HR, 0.78; 95% CI, 0.70-0.86; *p <*0.001) or rectum (HR, 0.86; 95% CI, 0.80-0.92; *p <*0.001) showed better survival, while appendiceal early-onset GEP-NENs demonstrated the worse OS (HR, 1.30; 95% CI, 1.21-1.39; *p <*0.001).

## Discussion

Given the prevalence of early-onset cancer and the extension of lifespan, heightened attention must be paid to early-onset cancers (22–24). The incidence of GEP-NENs has been increasing for decades worldwide (20). Currently, more studies tend to focus on GEP-NENs, and few studies have focused on early-onset GEP-NENs. Here, we conducted the largest population-based epidemiological study of early-onset GEP-NENs from 1975 to 2018, and illustrated the incidence, mortality trends, and survival for the first time.

Our analysis indicated a notable rise in the incidence of early-onset GEP-NENs over the past few decades, surpassing the rate of increase observed for later-onset GEP-NENs. The factors driving these escalating trends might be multifaceted. Approximately 5% of GEP-NENs occur in the setting of inherited tumor syndromes, in which patients usually present at an earlier age at onset (under the age of 40). The main genetic syndromes include multiple endocrine neoplasia type 1 and multiple endocrine neoplasia type 4, associated with GEP-NENs; von Hippel-Lindau syndrome, tuberous sclerosis, neurofibromatosis type 1, and glucagon cell hyperplasia neoplasia, associated with pancreatic NENs (25–27). Besides, studies have suggested that a family history of cancer is the most relevant risk factor for NEN development (28). Neklason et al. found that the siblings and parents of patients with small intestine NENs have a 13.4-fold and 6.5-fold higher relative risks of developing GEP-NENs, respectively, compared to controls (29). In a population-based study that included 108 patients with early-onset lung or digestive NENs, 15.7% of patients had germline pathogenic variants, and these patients more often had a family history of cancer (30). Significant advancements have occurred in genotyping, genetic counselling, family screening, and various relevant biomarkers developing over the past years (31). Moreover, individuals carrying certain cancer predisposing genes have an increased chance of reproducing and passing variants to the next generation owing to advances in medicine (1). The aforementioned factors may contribute to an elevated prevalence of inherited tumor syndromes among young adults, ultimately leading to an increased occurrence of GEP-NENs in the young age group.

Although genetic factors play an important role in early-onset GEP-NENs, it must be acknowledged that the majority of patients have sporadic disease, which means that environmental and behavioral factors might also play a role in the increasing incidence. Obesity and diabetes are consistently recognized as risk factors for site-specific GEP-NENs (32, 33). Worldwide trends revealed that the prevalence of obesity among adults increased from 3.2% to 10.8% for men, and from 6.4% to 14.9% for women during 1975–2014 (34). Similarly, the prevalence of diabetes among children and adults has increased in multiple countries over the past several decades (35, 36). In addition, smoking and alcohol consumption were positively associated with the development of pancreatic NENs, whereas smoking only and alcohol only were associated with small intestine and rectal NENs, respectively (36, 37). Globally, the prevalence of smoking among individuals aged 15 years and older was 32.7% among males and 6.62% among females (38). Of the 1.34 billion individuals who consumed alcohol in 2020, 59.1% were 15 to 39 years old (39). Taken together, these data suggest that changes in environment and lifestyles are possible causes of the shift in the burden of GEP-NENs to the younger population.

A previous US study reported that the incidence of GEP-NENs in the rectum showed the most significant increase among site groups (21). However, in our study, we found that the increase was greatest in the stomach, which may be attributed to the increased availability of routine monitoring and endoscopy in clinical practice (40). The incidence increased across all stages except the unknown stage, consistent with other studies (8), which may be associated with advancements in imaging, the adoption of standardized staging system and pathology guidelines, and increased awareness of NENs.

Current evidence suggests that clinical and tumor characteristics exhibit differences among certain early-onset and later-onset cancer types (1). Shig et al. found that early-onset prostate cancer was resistant to androgen-deprivation therapy and had a poorer survival outcome (41). Early-onset colorectal cancer is more likely to be diagnosed at advanced disease stages and display aggressive tumor phenotypes (42). In this study, our investigation revealed that the clinical characteristics exhibited differentials among early-onset and later-onset GEP-NENs. In contrast to later-onset GEP-NEN, early-onset GEP-NEN demonstrated a higher incidence of female gender, well-differentiated tumor, and localized disease. In addition, the survival of patients with early-onset GEP-NENs was better than that of patients with later-onset GEP-NENs, regardless of the tumor site, grade, or stage. We further explored the factors that were independently associated with the risk of survival. The multivariable Cox proportional hazards regression analysis revealed that race, marital status, grade, stage, chemotherapy, and primary site were significantly associated with the survival of early-onset GEP-NENs patients. The above evidence suggests that early-onset and late-onset GEP-NENs might have differential mechanisms of carcinogenesis, and in-depth investigations on the molecular pathology, genomics, and multi-omics features of early-onset GEP-NENs could enhance the understanding of the etiologies and guide strategies for the prevention, early detection and treatment of both early-onset and later-onset GEP-NENs.

Our study also analyzed the incidence-based mortality for early-onset GEP-NENs, and found that it has continued to increase over the study period. Sun et al. reported that the highest cumulative mortality of GEP-NENs was caused by primary cancer during the early follow-up period, whereas that of other cancers and noncancer diseases exceeded that of primary cancer at approximately 90 and 170 months after diagnosis, respectively (43). In our study, the cumulative mortality of early-onset GEP-NENs was the highest across all follow-up periods. This result highlights the importance of early detection and treatment for early-onset GEP-NENs.

The current study has several limitations. First, selection bias was not avoided in a retrospective and population-based study. Second, benign tumors are not captured by the SEER database, which could potentially lead to an underestimation of the true incidence of early-onset GEP-NENs. Third, the absence of information pertaining to the Ki-67 index, morphological characteristics, novel precise grading, and chemotherapy regimen within the SEER database could potentially limit the generalizability of the findings. Finally, we acknowledge the limitation associated with employing a binary age classification at 50 years, and heterogeneity within this group should be considered. The optimal screening and treatment approaches for various age groups should be customized to the organ site as the age at cancer diagnosis can vary across different organ sites. Nonetheless, the cut-off value of 50 years was made to ensure uniformity in the collection and interpretation of current evidence pertaining to early-onset cancers.

## Conclusions

In summary, both the incidence and incidence-based mortality rates of early-onset GEP-NENs have continued to grow over the years, and rates of increase were greater than later-onset GEP-NENs. Genetic alterations, environmental factors, and lifestyle factors are possible causes driving these escalating trends. The clinical characteristics and survival exhibit differences among patients with early-onset and later-onset GEP-NENs. Race, marital status, grade, stage, chemotherapy, and primary site were identified as independent prognostic factors for early-onset GEP-NENs. Future studies on etiologic factors are warranted to better understand the survival and increasing incidence disparities between early-onset and later-onset GEP-NENs.

## Data availability statement

Publicly available datasets were analyzed in this study. This data can be found here: www.seer.cancer.gov.

## Ethics statement

Data are publicly available and do not require ethical approval for analysis. Authors obtained Limited-Use Data Agreements from SEER.

## Author contributions

HY: Data collection, analysis and wrote the manuscript. GH: Data collection, analysis and wrote the manuscript. HS: Designed and supervised the study. RL: Designed and supervised the study. CJ: Data collection and analysis. MF: Data collection and analysis. LY: Advised on analyses. The work reported in the paper has been performed by the authors, unless clearly specified in the text. All authors contributed to the article and approved the submitted version.

## Supplementary material

Supplementary Figure 1
**(A–D)** The overall survival between early-onset GEP-NENs and later-onset GEP-NENs groups by stage, **(A)** localized, **(B)** regional, **(C)** distant, **(D)** unknown stage. **(E–H)** The overall survival between early-onset GEP-NENs and later-onset GEP-NENs groups by grade, **(E)** grade I, **(F)** grade II, **(G)** grade III+IV, **(H)** unknown grade.
