# Supplementary figures and images for: Epidemiologic trends and survival of early-onset gastroenteropancreatic neuroendocrine neoplasms
Source: Front Endocrinol (Lausanne). 2023 Aug 28;14:1241724. doi: 10.3389/fendo.2023.1241724 (PMC10493410; doi:10.3389/fendo.2023.1241724)

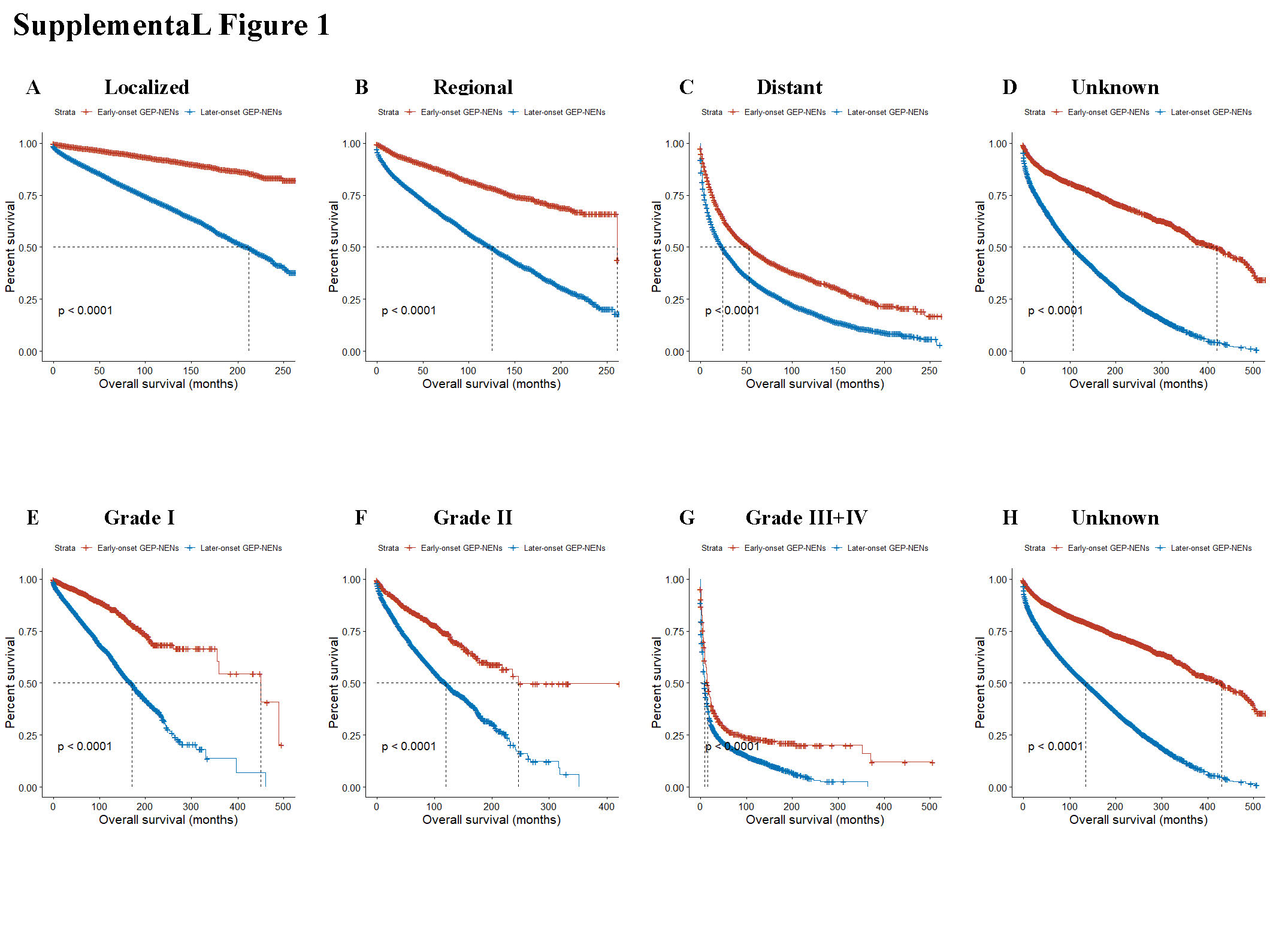

Supplement: Supplementary Figure 1 — (A–D) The overall survival between early-onset GEP-NENs and later-onset GEP-NENs groups by stage, (A) localized, (B) regional, (C) distant, (D) unknown stage. (E–H) The overall survival between early-onset GEP-NENs and later-onset GEP-NENs groups by grade, (E) grade I, (F) grade II, (G) grade III+IV, (H) unknown grade. [file Image_1.jpeg]
