# Supplementary material for: Epidemiologic trends and survival of early-onset gastroenteropancreatic neuroendocrine neoplasms
Source: Front Endocrinol (Lausanne). 2023 Aug 28;14:1241724. doi: 10.3389/fendo.2023.1241724 (PMC10493410; doi:10.3389/fendo.2023.1241724)
Supplement: Supplementary file 2 [file Table_1.docx]

**Supplemental Table 1. Histologic *ICD* Codes Used to Identify Gastroenteropancreatic Neuroendocrine Tumors**

| **NEN Histology** | ***ICD* codes** |
| --- | --- |
| 8013/3 | Large cell neuroendocrine carcinoma |
| 8150 | Pancreatic endocrine tumor |
| 8151/3 | Insulinoma |
| 8152/3 | Glucagonoma |
| 8153/3 | Gastrinoma |
| 8154/3 | Mixed pancreatic endocrine and exocrine tumor |
| 8155/3 | VIPoma |
| 8156/3 | Somatostatinoma |
| 8240 | Carcinoid tumor |
| 8241/3 | Enterochromaffin cell carcinoid |
| 8242/3 | Enterochromaffin-like cell tumors |
| 8243 | Goblet cell carcinoid |
| 8244/3 | Mixed adenoneuroendocrine carcinoid |
| 8245/3 | Adenocarcinoid tumor |
| 8246 | Neuroendocrine carcinoma |
| 8249/3 | Atypical carcinoid tumor |
| 8574 | Adenocarcinoma with neuroendocrine differentiation |
| 9091/3 | Stromal carcinoid |

Abbreviations: *ICD*, *International Classification of Diseases.*

**Supplemental Table 2. Survival Analysis of Patients with Early-Onset Gastroenteropancreatic Neuroendocrine Tumors Diagnosed from 1975 to 2018 by SEER Stage and Primary Site**

| **Primary Site** | **Median Survival (months)** | | |
| --- | --- | --- | --- |
|  | Localized | Regional | Distant |
| Appendix | NR | NR | 38 |
| Colon | NR | NR | 13 |
| Small intestine | NR | NR | 174 |
| Stomach | NR | NR | 14 |
| Rectum | NR | 147 | 15 |
| Pancreas | NR | 261 | 41 |

Abbreviations: NR, Not reach.

**Supplemental Table 3. Univariate Regression Analysis for Early-Onset** **Gastroenteropancreatic Neuroendocrine Tumors**

| **Characteristics** | **HR** | **95% CI** | ***p* value** |
| --- | --- | --- | --- |
| **Sex** |  |  |  |
| Female | 1 (Reference) | 1 (Reference) |  |
| Male | 0.95 | 0.92, 0.98 | 0.003 |
| **Race** |  |  |  |
| White | 1 (Reference) | 1 (Reference) |  |
| Black | 0.95 | 0.91, 1.00 | 0.048 |
| AI/AP | 1.01 | 0.95, 1.07 | 0.862 |
| Unknown | 1.71 | 1.57, 1.86 | <0.001 |
| **Marital status** |  |  |  |
| Married | 1 (Reference) | 1 (Reference) |  |
| Single | 1.41 | 1.36, 1.46 | <0.001 |
| Sep/Div/Wid | 1.30 | 1.21, 1.39 | <0.001 |
| Unknown | 1.10 | 1.04, 1.16 | <0.001 |
| **SEER stage** |  |  |  |
| Localized | 1 (Reference) | 1 (Reference) |  |
| Regional | 0.95 | 0.91, 1.00 | 0.039 |
| Distant | 0.71 | 0.67, 0.76 | <0.001 |
| Unknown | 0.19 | 0.17, 0.20 | <0.001 |
| **Grade** |  |  |  |
| I | 1 (Reference) | 1 (Reference) |  |
| II | 0.83 | 0.77, 0.89 | <0.001 |
| III+IV | 0.41 | 0.36, 0.48 | <0.001 |
| Unknown | 0.47 | 0.46, 0.49 | <0.001 |
| **Surgery** |  |  |  |
| Yes | 1 (Reference) | 1 (Reference) |  |
| No | 1.69 | 0.24, 12.1 | 0.599 |
| Unknown | 0.95 | 0.64, 1.41 | 0.792 |
| **Chemotherapy** |  |  |  |
| Yes | 1 (Reference) | 1 (Reference) |  |
| No/Unknown | 1.31 | 1.20, 1.42 | <0.001 |
| **Radiation** |  |  |  |
| Yes | 1 (Reference) | 1 (Reference) |  |
| No/Unknown | 1.41 | 1.18, 1.69 | <0.001 |
| **Primary site** |  |  |  |
| Stomach | 1 (Reference) | 1 (Reference) |  |
| Small Intestine | 0.84 | 0.78, 0.90 | <0.001 |
| Colon | 0.70 | 0.63, 0.77 | <0.001 |
| Appendix | 1.36 | 1.28, 1.46 | <0.001 |
| Rectum | 0.87 | 0.82, 0.93 | <0.001 |
| Pancreas | 1.05 | 0.97, 1.13 | 0.220 |
| Abbreviations: HR, Hazard Ratio; CI, Confidence Interval; AI/AN, American Indian/Alaska Native; AP, Asian or Pacific Islander; Sep/Div/Wid, Separated/ Divorced/ Widowed. | | | |
